# Dataset on mobile video game engagement, gamer experience, and behavioral intentions among Indonesian gamers in a transient educational hub using PLS-SEM

**DOI:** 10.1016/j.dib.2026.113212

**Published:** 2026-08-27

**Authors:** Fyona Chelindiva, Vita Megawati, Laurence

**Affiliations:** a Faculty of Economics and Business, Universitas Pelita Harapan; b Industrial Engineering Department, Faculty of Science and Technology, Universitas Pelita Harapan

**Keywords:** Video game engagement, Gamer experience, Arousal, Enjoyment, Behavioral intention, PLS-SEM, SmartPLS 4, Mobile gaming

## Abstract

This paper presents a dataset examining mobile video game engagement and gamer experience among Indonesian gamers residing in a transient educational hub, Kampung Inggris, Pare, Kediri, East Java. Data were collected through an online questionnaire distributed to 202 valid respondents belonging to the Millennial (born 1981–1996) and Generation Z (born 1997–2012) cohorts who had actively played video games within the past year. The dataset captures thirteen constructs: Telepresence (TP), Focused Attention (FA), Role Projection (RP), Fantasy Fulfillment (FF), Emotional Involvement (EI), Enjoyment (EJ), Arousal (AR), Gamer Experience (GE), Video Game Engagement (VGE), Intention to Continue Playing (ICP), Intention to Purchase Game Items (IPI), Intention to Engage in Word-of-Mouth (IWOM), and Intention to Recruit New Players (IRN), measured across 72 Likert-scale indicators. Analysis was performed using Partial Least Squares Structural Equation Modeling (PLS-SEM) with SmartPLS 4. The dataset package includes raw survey data, a cleaned PLS-ready input file, SmartPLS algorithm output, bootstrapping results, Importance-Performance Map Analysis (IPMA) output, PLSpredict results, questionnaire items, English and Bahasa Indonesia versions of the questionnaire, and informed consent. This dataset is a resource for researchers investigating affect-related engagement, behavioral intentions, and social gaming dynamics within mobile and peer-concentrated digital environments.

Specifications Table

**Table:** 

Subject	Social Sciences
Specific subject area	Digital gaming behavior, mobile game engagement, affect-related behavioral intentions, and consumer behavior in digital entertainment environments.
Type of data	Analyzed; Raw; Filtered; Processed. File formats: (a) 01_Raw_Dataset_202_Respondents.xlsx: raw Google Forms survey data - demographic profiles and 72 Likert-scale responses. (b) 02_SmartPLS_Ready_Dataset_202_Respondents.xlsx: cleaned dataset formatted for SmartPLS 4 analysis, containing only measurement indicators (202 respondents × 72 indicators). (c) 03_PLS_SEM_Algorithm_Output.xlsx: SmartPLS 4 algorithm output including outer loadings, path coefficients, R², f², HTMT, model fit (SRMR). (d) 04_Bootstrapping_Results.xlsx: bootstrapping results (5,000 subsamples) including T-statistics, p-values, bias-corrected confidence intervals. (e) 05_IPMA_Results.xlsx: Importance-Performance Map Analysis output. (f) 06_PLSpredict_Results.xlsx: PLSpredict output including Q² predict, RMSE, MAE (PLS-SEM vs. linear model benchmark). (g) 07_Questionnaire_Items_and_Measurement_Scale.xlsx: Questionnaire codebook containing construct names, indicator codes, questionnaire item numbers, and the corresponding Likert-scale measurement. (h) 08_English_Questionnaire_and_Informed_Consent.pdf: English-language version of the full 88-item questionnaire and informed consent form. (i) 09_Indonesian_Questionnaire_and_Informed_Consent.pdf: Bahasa Indonesia version of the full 88-item questionnaire and informed consent form. (j) 10_Statement_Regarding_Participant_Eligibility.pdf: statement documenting participant eligibility screening criteria and procedure.
Data collection	An online questionnaire via Google Forms was distributed to active video game players residing in Kampung Inggris, Pare, Kediri, East Java, Indonesia, a transient English immersion community that attracts students from diverse provinces across Indonesia. Purposive sampling was employed; eligibility required respondents to have been born between 1981 and 2012 (covering the Millennial and Generation Z cohorts) and to have actively played video games within the past year. A five-point Likert scale (1 = Strongly Disagree; 5 = Strongly Agree) was applied across all 72 measurement items. The survey link was disseminated primarily via WhatsApp groups maintained by English-course communities in Pare, supplemented by direct peer referral (snowball sampling). A total of 202 valid responses were retained after screening.
Data source location	Country: Indonesia; Area/City/Region: Kampung Inggris, Pare, Kediri, East Java. Note: Although data collection was localized to this site, respondents originate from multiple provinces across Indonesia, making the sample geographically diverse at the national level.
Data accessibility	Repository name: Mendeley Data Data identification number: 10.17632/46np2n5cc6.3 Direct URL to data: https://data.mendeley.com/datasets/46np2n5cc6/3 for accessing these data: openly accessible via the DOI/URL above; no special permissions required.
Related research article	None

## 1 Value of the Data

• This dataset provides a multidimensional empirical resource for investigating how gamer experience dimensions and affective states are associated with video game engagement and downstream behavioral intentions among mobile gamers embedded in a transient educational hub in Indonesia. The dataset supports analysis of both the full thirteen-construct model and simplified sub-models, offering flexibility for diverse research designs.
• Researchers can reuse this dataset to examine the structural relationships between experiential antecedents (Telepresence, Focused Attention, Role Projection, Fantasy Fulfillment, Emotional Involvement, Enjoyment, and Arousal), holistic Gamer Experience, Video Game Engagement, and behavioral outcomes (continuation, in-game purchase, word-of-mouth advocacy, and peer recruitment). The data also support predictive modeling, mediation analysis, and importance-performance mapping.
• The dataset captures a geographically diverse convenience sample drawn from a transient educational hub that attracts participants from multiple provinces. This sampling context enables secondary analyses related to peer influence, social diffusion of gaming behavior, and the intersection of leisure practices and short-term collective living.
• Game developers, digital marketers, and community managers may use this dataset to explore which affective factors are most strongly associated with player engagement within competitive mobile gaming contexts, and to inform future engagement and communication strategies.
• This dataset may be useful for researchers conducting comparative studies in different gaming contexts or populations, as well as for evaluating and extending existing PLS-SEM models. The dataset should be interpreted in the context of its cross-sectional design and non-probability sampling approach. It is suitable for examining associations among the study constructs, while conclusions regarding causality or broader population generalization should be made with caution.

## 2 Background

Indonesia’s mobile gaming sector represents one of Southeast Asia’s most dynamic digital entertainment markets, characterized by an expansive base of young, socially connected players who engage predominantly through smartphones [1]. As competition intensifies across gaming platforms, sustaining player engagement has become a central strategic priority for game developers, shifting the focus of both industry and academic inquiry from adoption to retention and social amplification behaviors [2].

Within this evolving landscape, gamer experience has emerged as a theoretically central construct capturing the holistic psychological evaluations players form through interactions with game systems. Prior scholarship has conceptualized gamer experience as an integrative appraisal process spanning emotional, cognitive, and sensory dimensions [3]. While earlier frameworks emphasized narrative immersion, role projection, and fantasy fulfillment as key experiential dimensions, contemporary evidence suggests that immediate affective states, particularly enjoyment and arousal, may assume greater salience in fast-paced, mobile-first, and session-based gaming environments [4,5].

Enjoyment, reflecting hedonic pleasure and intrinsic satisfaction derived from gameplay, has been consistently associated with positive experiential evaluations and subsequent engagement [6]. Arousal, capturing physiological and psychological activation, excitement, and heightened alertness during play, has been associated with more positive experiential evaluations, particularly when gameplay mechanics demand rapid decision-making and competitive performance [5]. Together, these affective dimensions represent important aspects of gamer experience and are theorized to be associated with players’ broader evaluations of gameplay quality.

Video Game Engagement (VGE), in turn, represents a state of sustained psychological investment encompassing cognitive absorption, emotional attachment, and behavioral activation [7]. Engagement has been associated with behavioral intentions, including continuance intention, in-game purchasing, word-of-mouth advocacy, and peer recruitment [8].

This dataset was developed to address the limited availability of datasets examining the associations among gamer experience, engagement, and behavioral intentions in a socially dense and transient youth ecosystem in Indonesia. Using PLS-SEM [9] implemented in SmartPLS 4 [10], the dataset enables examination of both explanatory and predictive dimensions of the proposed model.

## 3 Data Description

### 3.1 Dataset location and description

The complete dataset is archived in the Mendeley Data repository [11]. It comprises ten interlinked files corresponding to successive stages of the research workflow, from raw data collection through to advanced PLS-SEM diagnostics, enabling full replication of the reported analyses and supporting independent secondary research.

(a) ***01_Raw_Dataset_202_Respondents.xlsx:*** This file contains the original questionnaire responses exported from Google Forms after eligibility screening. Only respondents who met the study inclusion criteria (belonging to the target generational groups and reporting that they played mobile video games) were retained, resulting in 202 valid responses. Questions 1–16 include demographic and gaming-related information, such as generational cohort, gender, occupation, education level, gaming expenditure, payment method for in-game purchases, gaming device, preferred game, gaming duration, years of gaming experience, and gaming companions. Questions 17–88 contain responses to the 72 Likert-scale items measuring Telepresence, Focused Attention, Role Projection, Fantasy Fulfillment, Emotional Involvement, Enjoyment, Arousal, Gamer Experience, Video Game Engagement, Intention to Continue Playing, Intention to Purchase Game Items, Intention to Engage in Word-of-Mouth, and Intention to Recruit New Players. The questionnaire structure and original question numbering were retained, making this file suitable for descriptive analyses, demographic profiling, and further data preparation.
(b) ***02_SmartPLS_Ready_Dataset_202_Respondents.xlsx:*** This file contains the same 202 screened responses reorganized for analysis in SmartPLS 4. The original questionnaire layout was converted into a construct-based dataset, with the 72 measurement items arranged according to the thirteen latent constructs (TP1–TP6, FA1–FA6, RP1–RP4, FF1–FF7, EI1–EI5, EJ1–EJ4, AR1–AR7, GE1–GE7, VGE1–VGE7, ICP1–ICP4, IPI1–IPI5, IWOM1–IWOM5, and IRN1–IRN5). Variable names follow the SmartPLS 4 indicator naming convention, allowing the dataset to be imported directly without additional preprocessing.
(c) ***03_PLS_SEM_Algorithm_Output.xlsx:*** The complete SmartPLS 4 algorithm output for the full thirteen-construct model, including direct, indirect, and total path coefficients; outer loadings and outer weights; R-squared and adjusted R-squared values; f-squared effect sizes; construct reliability and validity statistics (Cronbach’s Alpha, rho_a, Composite Reliability, and AVE); HTMT discriminant validity ratios; model fit indices (SRMR, NFI, Chi-square); and standardized latent variable scores for all 202 cases.
(d) ***04_Bootstrapping_Results.xlsx:*** This file contains SmartPLS 4 bootstrapping results derived from 5000 subsamples. For all path coefficients, indirect effects, and outer loadings, this file provides the original sample estimate, bootstrap mean, standard deviation, T-statistic, p-value, and bias-corrected confidence intervals (2.5th and 97.5th percentiles). Results are organized into direct effects, total indirect effects, and specific indirect effects sub-sections.
(e) ***05_IPMA_Results.xlsx:*** This file presents the Importance-Performance Map Analysis (IPMA) output from SmartPLS 4. The file reports total effect values (importance) and rescaled performance indices (0–100 scale) for each predictor construct with respect to the designated target construct, enabling identification of constructs with high strategic priority.
(f) ***06_PLSpredict_Results.xlsx:*** This file contains PLSpredict output providing indicator-level predictive accuracy metrics, including Q² predict values, Root Mean Square Error (RMSE), and Mean Absolute Error (MAE) for both the PLS-SEM model and a linear model benchmark. These outputs allow readers to compare the predictive performance of the proposed model with that of a benchmark linear model.
(g) ***07_Questionnaire_Items_and_Measurement_Scale.xlsx:*** This file provides a codebook for all questionnaire items used in the study. It includes the question number, item code, construct, questionnaire statement, and response scale for each measurement item.
(h) ***08_English_Questionnaire_and_Informed_Consent.pdf:*** This file contains the English translation of the original Bahasa Indonesia survey instrument and informed consent form. It includes the same 88 questionnaire items (16 demographic and gaming-related questions and 72 Likert-scale measurement items).
(i) ***09_Indonesian_Questionnaire_and_Informed_Consent.pdf:*** The Bahasa Indonesia version of the same 88-item survey instrument and informed consent form used for data collection, It includes all 88 questionnaire items administered to participants.
(j) ***10_Statement_Regarding_Participant_Eligibility.pdf:*** A statement documenting the eligibility screening criteria applied to respondents before data analysis.

### 3.2 Data collection

Survey data were gathered through an online questionnaire administered via Google Forms. Recruitment was conducted within Kampung Inggris, Pare, Kediri, East Java, Indonesia, a nationally recognized English immersion hub that draws students from provinces across the country for short-term intensive language programs [13].

Purposive sampling was applied to ensure that all participants satisfied two eligibility criteria: (1) birth between 1981 and 2012 (covering the Millennial and Generation Z cohorts), and (2) active video game playing experience within the recent period. Respondents were informed of the study’s academic purpose, voluntary nature, data confidentiality provisions, and their right to withdraw at any stage. No personally identifiable information was collected. To supplement the initial purposive approach, snowball sampling was employed, with participants encouraged to refer other eligible gamers within their immediate social networks.

The questionnaire comprised 88 items organized into two sections. The first section (Questions 1–16) captured demographic and gaming behavioral characteristics including generational cohort, gender, current occupation, educational background, primary gaming device, gaming frequency, average daily play duration, gaming tenure, usual playing companions, and monthly in-game spending. The second section (Questions 17–88) consisted of 72 Likert-scale items rated on a five-point scale (1 = Strongly Disagree; 5 = Strongly Agree), measuring the thirteen study constructs. The original questionnaire was prepared in Bahasa Indonesia and used for data collection. Google Forms was configured to require responses to all questionnaire items, resulting in no missing values in the final dataset.

### 3.3 Descriptive data analysis

The final analytical dataset comprised 202 complete responses. No imputation procedures were necessary. Table 1 presents the demographic and gaming profile of the final sample.

**Table 1:** Respondent profile (N = 202).

Characteristic	n	%
**Gender**		
Male	106	52.5
Female	96	47.5
**Generation**		
Generation Z (born 1997–2012)	145	71.8
Millennial (born 1981–1996)	57	28.2
**Last Educational Background**		
Senior High School / Vocational (SMA/SMK)	123	60.9
Bachelor’s Degree (S1)	58	28.7
Diploma (D3/D4)	8	4.0
Master’s Degree (S2)	9	4.5
Doctoral Degree (S3)	4	2.0
**Job Status**		
High School Student (Siswa)	68	33.7
University Student (Mahasiswa)	49	24.3
Private Employee	27	13.4
Not Working	20	9.9
Others (Entrepreneur, Part-timer, Freelance, etc.)	38	18.8
**Primary Gaming Device**		
Mobile Phone	168	83.2
Computer/PC	24	11.9
Others (Console, Tablet)	10	4.9
**Gaming Frequency (days/week)**		
Daily (7 days)	48	23.8
Frequent (4–6 days)	43	21.3
Occasional (1–3 days)	111	54.9
**Gaming Experience Duration**		
Long-term (> 3 years)	74	36.6
Mid-term (1–3 years)	78	38.6
Novice (< 1 year)	47	23.3
Others / Unspecified	3	1.5
**Monthly Spending on In-Game Items**		
None	86	42.6
IDR ≤ 50,000	42	20.8
IDR 51,000 – 100,000	22	10.9
IDR 101,000 – 200,000	26	12.9
IDR 201,000 – 500,000	16	7.9
Above IDR 500,000	10	5.0

Table 1 shows that Generation Z respondents accounted for 71.8% of the final sample. The near-equal gender distribution (52.5% male, 47.5% female) provides a balanced demographic composition within the sampled respondents. High school and university students accounted for 58.0% of the final sample. Mobile phones were the dominant gaming platform (83.2%) among respondents [14]. A substantial proportion of respondents (75.2%) have accumulated between one and more than three years of gaming experience. Approximately 57.4% of respondents reported in-game spending, with the majority confined to lower expenditure tiers.

### 3.4 Outlier detection analysis

**Fig. 1:**
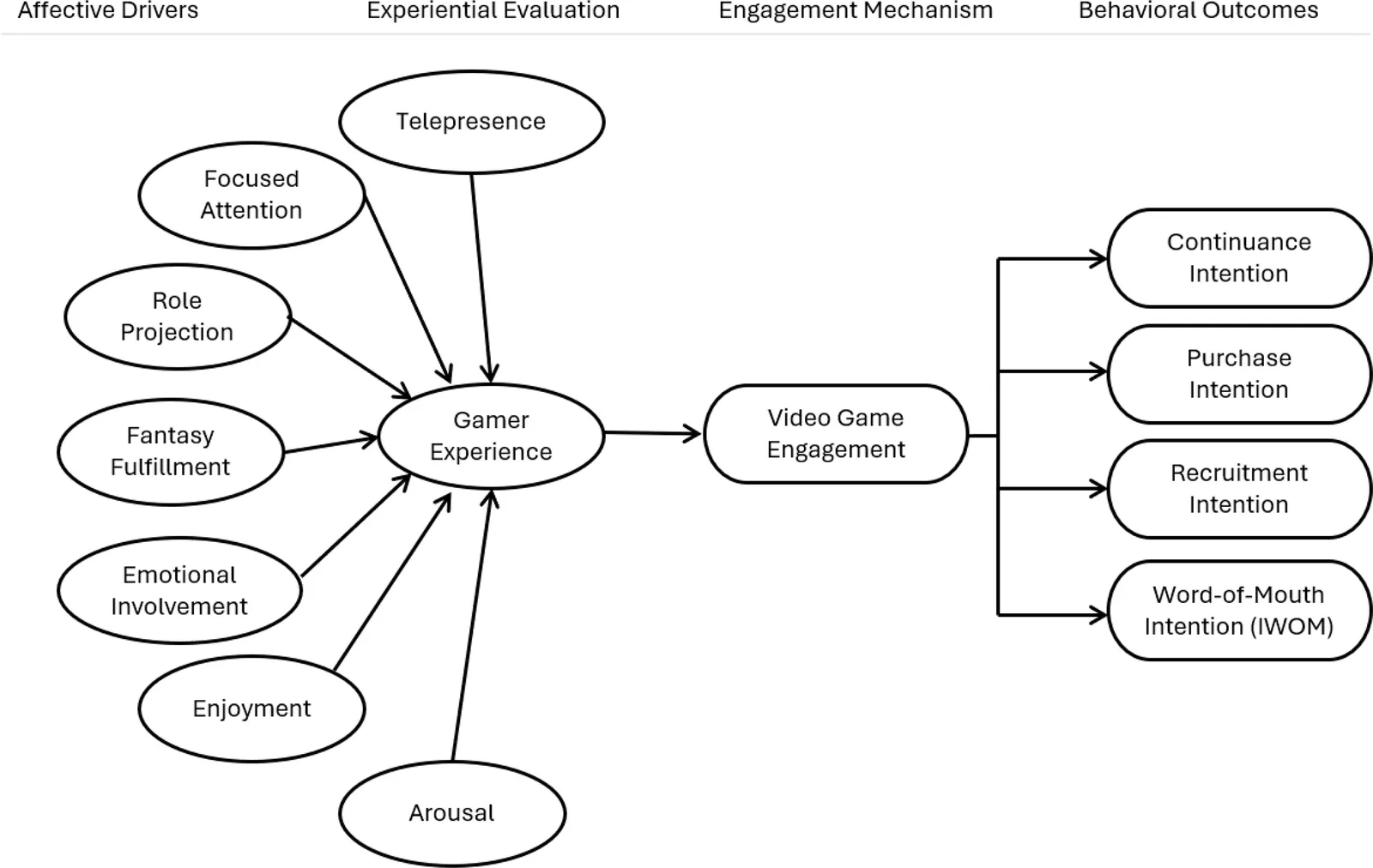
Research Model.

Prior to structural analysis, response quality was assessed through straight-lining detection, identifying respondents who selected an identical response across a disproportionate number of items, which may indicate disengaged or invalid responding. Of the 220 responses initially collected, 18 exhibited straight-lining response patterns and were excluded, resulting a final analytical dataset of 202 valid responses. Univariate outlier detection was subsequently conducted using standardized Z-scores. Consistent with the guideline that, for samples exceeding 80 observations, a Z-score of ±4.0 constitutes the threshold for classifying an extreme outlier [15] no data points across any of the thirteen constructs exceeded this criterion. The final analytical dataset of 202 cases was therefore retained in its entirety without further exclusion (Fig. 1).

### 3.5 Data analysis

Structural analysis was conducted using PLS-SEM implemented in SmartPLS 4 [10]. PLS-SEM was selected for its suitability in handling complex, multi-construct models with latent variables, its robustness to distributional assumptions, and its capacity to simultaneously evaluate both explanatory and predictive model dimensions [9].

The analytical procedure followed a two-stage approach. In Stage 1, the measurement model was evaluated using indicator outer loadings (threshold > 0.70), Cronbach’s Alpha and rho_a (> 0.70) for internal consistency reliability, Composite Reliability (rho_c > 0.70) for construct reliability, and Average Variance Extracted (AVE > 0.50) for convergent validity. Discriminant validity was assessed using the Heterotrait-Monotrait (HTMT) ratio criterion [16]. Table 2 presents the measurement model assessment results.

**Table 2:** Construct reliability and validity results.

Construct	Items	Cronbach’s Alpha	rho_a	CR (rho_c)	AVE
Telepresence (TP)	6	0.894	0.904	0.919	0.657
Focused Attention (FA)	6	0.888	0.897	0.914	0.640
Role Projection (RP)	4	0.897	0.903	0.928	0.765
Fantasy Fulfillment (FF)	7	0.920	0.924	0.936	0.676
Emotional Involvement (EI)	5	0.895	0.897	0.923	0.707
Enjoyment (EJ)	4	0.892	0.894	0.925	0.756
Arousal (AR)	7	0.902	0.911	0.923	0.634
Gamer Experience (GE)	7	0.921	0.922	0.937	0.679
Video Game Engagement (VGE)	7	0.888	0.898	0.913	0.603
Intention to Continue Playing (ICP)	4	0.917	0.917	0.941	0.800
Intention to Purchase Game Item (IPI)	5	0.931	0.935	0.948	0.784
Intention to Engage in WOM (IWOM)	5	0.905	0.910	0.929	0.724
Intention to Recruit New Player (IRN)	5	0.902	0.904	0.927	0.719

Note: CR = Composite Reliability (rho_c); AVE = Average Variance Extracted. All thresholds: Alpha > 0.70; rho_a > 0.70; CR > 0.70; AVE > 0.50.

As shown in Table 2, all thirteen constructs satisfy established thresholds for internal consistency, construct reliability, and convergent validity [9]. Discriminant validity was subsequently assessed via HTMT criterion, was supported for most construct pairs [16]. Seven construct pairs exceeded the 0.85 criterion. Three pairs slightly exceeded the more conservative 0.90 threshold: Intention to Recruit New Player with Intention to Engage in Word-of-Mouth (HTMT = 0.943), Arousal with Gamer Experience (HTMT = 0.916), and Enjoyment with Gamer Experience (HTMT = 0.904). A further four pairs fell between 0.85 and 0.90: Video Game Engagement with Gamer Experience (HTMT = 0.889), Video Game Engagement with Arousal (HTMT = 0.875), Role Projection with Fantasy Fulfillment (HTMT = 0.874), and Enjoyment with Arousal (HTMT = 0.854). Because seven HTMT values exceeded the recommended threshold, HTMT inference was performed to assess discriminant validity.

Bias-corrected bootstrap confidence intervals (5000 subsamples) were further examined for the seven construct pairs that exceeded the HTMT threshold. For all seven pairs, the upper bound of the 95% confidence remained below 1.0: Intention to Recruit New Player with Intention to Engage in Word-of-Mouth (95% CI [0.892, 0.985]), Arousal with Gamer Experience (95% CI [0.867, 0.959]), Enjoyment with Gamer Experience (95% CI [0.842, 0.957]), Video Game Engagement with Gamer Experience (95% CI [0.839, 0.934]), Video Game Engagement with Arousal (95% CI [0.819, 0.925]), Role Projection with Fantasy Fulfillment (95% CI [0.807, 0.930]), and Enjoyment with Arousal (95% CI [0.779, 0.921]). These results support acceptable discriminant validity for the measurement model under the HTMT inference criterion [16].

Because the data were collected via a single self-report questionnaire administered at one point in time, common method bias (CMB) was formally assessed using Harman’s single-factor test [17]. Harman's single-factor test was conducted using an unrotated principal component analysis of all 72 indicators in IBM SPSS Statistics, with Principal Component Analysis selected as the extraction method. The first extracted factor accounted for 44.7% of the total variance, which is below the 50% threshold for Harman's single-factor test [18]. The results are presented in (Tables 2b and 3)

**Table 2b:** Harman’s single-factor test: total variance explained (first 10 of 72 components).

Component	Eigenvalue	% of Variance	Cumulative %
1	32.180	44.695%	44.695%
2	5.014	6.964%	51.659%
3	4.540	6.305%	57.964%
4	2.332	3.239%	61.203%
5	1.762	2.448%	63.651%
6	1.308	1.817%	65.468%
7	1.244	1.728%	67.196%
8	1.209	1.679%	68.874%
9	1.122	1.558%	70.433%
10	1.069	1.485%	71.917%

**Table 3:** Structural model assessment: path coefficients.

Structural Path	β	STDEV	T-Statistic	p-value
Arousal (AR) → Gamer Experience (GE)	0.482	0.090	5.335	< 0.001***
Emotional Involvement (EI) → GE	0.004	0.062	0.072	0.942
Enjoyment (EJ) → Gamer Experience (GE)	0.391	0.069	5.676	< 0.001***
Fantasy Fulfillment (FF) → GE	0.071	0.070	1.016	0.310
Focused Attention (FA) → GE	0.058	0.058	1.000	0.317
Role Projection (RP) → GE	0.046	0.062	0.737	0.461
Telepresence (TP) → GE	−0.115	0.060	1.913	0.056
Gamer Experience (GE) → Video Game Engagement (VGE)	0.811	0.025	32.534	< 0.001***
VGE → Intention to Continue Playing (ICP)	0.683	0.051	13.452	< 0.001***
VGE → Intention to Engage in WOM (IWOM)	0.697	0.048	14.661	< 0.001***
VGE → Intention to Purchase Game Item (IPI)	0.617	0.050	12.455	< 0.001***
VGE → Intention to Recruit New Player (IRN)	0.620	0.053	11.669	< 0.001***

Note: *** p < 0.001; bootstrapping with 5,000 subsamples; β = path coefficient; STDEV = standard deviation; VGE = Video Game Engagement.

Table 4 presents the coefficients of determination (R²) for the endogenous constructs. The seven predictor constructs explained 78.5% of the variance in Gamer Experience and 65.8% of the variance in Video Game Engagement [15]. R² values for the four behavioral intention constructs ranged from 0.381 to 0.485, with IWOM exhibiting the highest value. Model fit was confirmed through the Standardized Root Mean Square Residual (SRMR = 0.066 for the saturated model), which falls below the recommended threshold of 0.08 [9], indicating adequate model fit.

**Table 4:** Coefficients of determination (R²).

Endogenous Construct	R²	R² Adjusted
Gamer Experience (GE)	0.785	0.777
Video Game Engagement (VGE)	0.658	0.657
Intention to Continue Playing (ICP)	0.467	0.464
Intention to Engage in WOM (IWOM)	0.485	0.483
Intention to Purchase Game Item (IPI)	0.381	0.378
Intention to Recruit New Player (IRN)	0.384	0.381

## 4 Experimental Design, Materials and Methods

### 4.1 Measurement instrument

The research instrument comprised thirteen validated, multi-item constructs administered on a five-point Likert scale. The measurement scales were adapted from established instruments in the digital gaming and consumer behavior literature and administered in Bahasa Indonesia. Table 5 provides a summary of each construct, its item count, representative questionnaire items, and source reference.

**Table 5:** Questionnaire items and measurement sources.

Code	Construct and Representative Items	Reference
TP1–TP6	Telepresence (6 items): Sense of physical presence and transportation within the game world (e.g., ‘I tend to lose awareness of my surroundings when playing’; ‘After playing, I feel as though I have returned from a journey’)	Laurence et al. [1]
FA1–FA6	Focused Attention (6 items): Cognitive absorption and time distortion during gameplay (e.g., ‘I do not think about other things while playing’; ‘I frequently lose track of time during sessions’)	Laurence et al. [1]
RP1–RP4	Role Projection (4 items): Identification with and projection into in-game characters and roles (e.g., ‘Playing enables me to project myself into a specific character’; ‘Playing allows me to take on a distinct persona’)	Hollebeek et al. [6]; Laurence et al. [1]
FF1–FF7	Fantasy Fulfillment (7 items): Engagement in imaginary experiences unavailable in everyday life (e.g., ‘Playing allows me to do things impossible in the real world’; ‘The game gives me freedom to explore worlds I can only imagine’)	Hollebeek et al. [6]; Laurence et al. [1]
EI1–EI5	Emotional Involvement (5 items): Depth of emotional connection with game narrative and world (e.g., ‘I feel deeply emotionally engaged while playing’; ‘I experience strong emotions when progressing through the game’)	Hammami [19]; Hollebeek et al. [6]
EJ1–EJ4	Enjoyment (4 items): Hedonic pleasure, fun, and intrinsic satisfaction derived from gameplay (e.g., ‘Playing provides me with genuine personal pleasure’; ‘I enjoy every gaming session I participate in’)	Hollebeek et al. [6]; Laurence et al. [1]
AR1–AR7	Arousal (7 items): Physiological and psychological activation during gameplay (e.g., ‘Playing increases my adrenaline level’; ‘I feel highly enthusiastic and energized during sessions’)	Juvrud et al. [5]; Hollebeek et al. [6]
GE1–GE7	Gamer Experience (7 items): Holistic evaluative appraisal of overall gameplay quality (e.g., ‘Playing introduces me to new strategies and techniques’; ‘The game content surprises and engages me’)	Laurence et al. [1]
VGE1–VGE7	Video Game Engagement (7 items): Depth of sustained psychological investment in the game (e.g., ‘This game is genuinely appealing to me’; ‘Playing this game consistently increases my interest’)	Laurence et al. [1]; Nadeem et al. [7]
ICP1–ICP4	Intention to Continue Playing (4 items): Forward-looking intentions to maintain gameplay over time (e.g., ‘I intend to continue playing this game rather than abandoning it’; ‘I plan to engage with this game over the long term’)	Hsiao & Chiou [2]
IPI1–IPI5	Intention to Purchase Game Items (5 items): Willingness to acquire virtual items within the game (e.g., ‘I plan to purchase virtual items in this game in the future’; ‘I have a strong intention to buy in-game items’)	Gibson et al. [20]; Putratama & Retnowardhani [14]
IWOM1–IWOM5	Intention to Engage in WOM (5 items): Advocacy and recommendation behaviors toward the game (e.g., ‘I intend to recommend this game to people in my network’; ‘I will post positive reviews about this game on digital platforms’)	Anubha et al. [21]
IRN1–IRN5	Intention to Recruit New Players (5 items): Active invitation of new individuals to join the gaming community (e.g., ‘I actively invite friends to try this game’; ‘I work to generate interest in this game among people who have not yet played it’)	Anubha et al. [21]; Jasrotia et al. [8]

The PLS-SEM analysis was executed in SmartPLS 4 [10] following the two-stage procedure outlined in Hair and Alamer [9]. Bootstrapping with 5,000 subsamples generated T-statistics and p-values for all path coefficients and indirect effects. Predictive relevance was assessed using PLSpredict [12], comparing PLS-SEM and linear model prediction errors via Q² predict, RMSE, and MAE. Importance-Performance Map Analysis (IPMA) was subsequently conducted to translate structural findings into managerially actionable priority rankings of predictor constructs.

### 4.2 Survey administration period

Data collection was conducted via an online, Google Forms-based questionnaire during November 2025. The survey link was distributed on a purposive and snowball basis, targeting active video game players temporarily residing in Kampung Inggris, Pare, Kediri, East Java. The primary distribution channel was a set of WhatsApp groups maintained by English-course communities in Pare, comprising participants enrolled in intensive language programs at various institutes in the area, predominantly young adults from the Generation Z and Millennial cohorts originating from multiple provinces across Indonesia. In addition to group-based distribution, snowball sampling was applied by encouraging participants to forward the survey link to fellow gamers within their personal networks. Google Forms was configured with mandatory responses on every item, ensuring no missing values across the collected responses.

### 4.3 Operational definition of straight-lining and exclusion criteria

Of the 220 initial responses, a data screening procedure was applied to detect straight-lining, defined as selecting the same response option across most of the 72 Likert-scale items. Responses with a standard deviation of zero were excluded because they showed no variation across all measurement items. Through this procedure, 18 responses were excluded, leaving 202 valid responses for further analysis. No missing-data imputation was required, as every item was mandatory in Google Forms.

### 4.4 Questionnaire adaptation and translation

The questionnaire was adapted from validated measurement scales originally published in English. The items were translated into Bahasa Indonesia before data collection and administered to all respondents in that language. Both the Bahasa Indonesia questionnaire and its English version are provided in the supporting data repository.

### 4.5 Variable codebook

The questionnaire comprises 72 Likert-scale items distributed across thirteen latent constructs. Each indicator was assigned a standardized alphanumeric code (e.g., TP1–TP6, FA1–FA6, and RP1–RP4) to ensure consistent identification across the dataset and SmartPLS analysis. To support secondary use of this dataset, a comprehensive Variable Codebook (07_Questionnaire_Items_and_Measurement_Scale.xlsx) is provided in the Mendeley Data repository. The file lists the variable name, questionnaire number, item code, questionnaire statement in English, and response scale for each measurement item. This documentation is intended to help users identify, interpret, and reference the variables included in the dataset.

## Limitations

The dataset was collected using purposive and snowball sampling within a single transient educational hub in Kampung Inggris, Pare, which may not represent gamers from other regions or settings. Although respondents originate from multiple provinces across Indonesia, the sample was not designed to represent the Indonesian gamer population as a whole. In addition, Generation Z respondents accounted for 71.8% of the sample, whereas Millennials represented 28.2%, meaning that younger gamers are more strongly represented in the dataset.

Data were collected at a single point in time (November 2025) using an online self-administered questionnaire. As a result, the dataset does not capture changes in gaming behavior or behavioral intentions over time. The responses were self-reported using five-point Likert scales and may therefore be affected by response bias, such as social desirability or recall bias. Age information was recorded as generational categories rather than exact ages, limiting analyses based on more detailed age differences. These considerations should be taken into account when reusing the data in future research.

## Ethics Statement

This study involved human subjects through a voluntary, non-invasive online survey. The research employed minimal-risk procedures with no experimental intervention, manipulation, or collection of personally identifiable or sensitive personal data, and was conducted in accordance with the principles of the Declaration of Helsinki. Because the study posed minimal risk and did not involve any intervention or sensitive data, it qualified for exemption from full ethical review. This exemption determination was reviewed and confirmed by the Ethics Review Committee of the Faculty of Economics and Business, Universitas Pelita Harapan (Reference No. SSHO-D-26-01772). Prior to completing the questionnaire, all participants received a clear explanation of the study’s academic purpose, data handling procedures, and their right to withdraw at any time without consequence. Informed consent was obtained, and responses were collected and analyzed in aggregate, fully anonymized form only. The authors confirm that they have read and follow the ethical requirements for publication in Data in Brief.

## Credit Author Statement

**Fyona Chelindiva**: Conceptualization, Methodology, Data curation, Formal analysis, Writing - Original Draft, Writing - Review & Editing; **Vita Megawati**: Methodology, Investigation, Resources, Writing - Original Draft, Writing - Review & Editing; **Laurence**: Supervision, Methodology, Formal analysis, Writing - review & editing.

## Data Availability

Mendeley DataDataset on Video Game Engagement and Gamer Experience among Indonesian Video Game Players using Partial Least Squares Structural Equation Model (PLS-SEM) (Original data).
